# Written or oral? The impact of social network relationships on land transfer contract choices: Evidence from the latest survey data in China

**DOI:** 10.1371/journal.pone.0335505

**Published:** 2025-11-13

**Authors:** Zexiao Sun, Zhonghan Wang

**Affiliations:** 1 School of Economics and Management, Hebei Agricultural University, Baoding, Hebei, China; 2 School of Public Policy and Administration, Xi’an Jiaotong University, Xi’an, Shaanxi, China; Policy Resaerch Institute, Government of Nepal, NEPAL

## Abstract

Facilitating the transfer of rural land is a significant initiative by the Chinese government aimed at optimizing the allocation of land resources and promoting agricultural modernization. Among these initiatives, encouraging farmers to establish written contracts is essential for maintaining order in agricultural land transfers and promoting large-scale land management. However, a substantial number of rural households in China still transfer land through oral agreements, and the underlying reasons behind this practice remain insufficiently understood. This study is grounded in social network theory and the theory of differential order. Utilizing questionnaire survey data from 636 rural households in North China collected in 2024, we examine the influence of social network relationships, such as kinship and geographical ties, on the choice of agricultural land transfer contracts. Our findings reveal that social network relationships significantly impact the choice of agricultural land transfer contracts. The closer the kinship and geographical ties, the more inclined farmers are to transfer land through oral agreements. Additionally, we uncover the moderating roles of farmers’ social trust and education level. Social trust amplifies the positive impact of social network relationships on the formation of oral agreements, while a higher level of education diminishes this effect. This paper provides a theoretical explanation for the widespread practice of oral contracting in rural land transfers in China. It also offers policy recommendations for the Chinese government.

## 1. Introduction

The high-quality development of agriculture underscores the importance of rational resource allocation, and a standardized land market can enhance the efficient utilization of land resources by improving transaction rules, reducing transaction costs, and minimizing information asymmetry, thereby boosting agricultural productivity [1]. According to relevant data, as of 2024, the area of cultivated land transferred in China has exceeded 555 million mu [2], indicating a continuous expansion trend in the country’s land transfer market. In the process of land marketization, standardized agricultural land transfers can facilitate rational resource allocation and improve land use efficiency. Compared to oral contracts, written contracts offer higher legal validity, stronger risk protection, greater transaction transparency, and better adaptability to complex situations. Written contracts can regulate the behavior of parties involved in land transfers and safeguard their rights and interests [3]. Currently, a significant number of farmers in China still opt for oral contracts in land transfers. According to rational choice theory, farmers are rational decision-makers aiming to maximize their utility or benefits. Given that written contracts can more effectively protect the rights and interests of both parties, and thus rational economic agents should logically prefer them. However, land transfers in rural China often occur within familiar social networks, where farmers establish a high degree of trust based on geographical and kinship ties. These trust-based social relationships and moral constraints serve as reputation signals and performance guarantees. Additionally, due to limited education, some farmers have an inadequate understanding of laws, regulations, and agricultural land transfer policies, making it difficult for written contracts to function as a safeguard.

Furthermore, oral contracts do not require complex written forms or legal procedures, resulting in lower transaction costs. Consequently, the use of oral contracts remains widespread, and the stability and legal validity of agricultural land transfer contracts still require further improvement. Exploring the factors influencing the choice of land transfer contracts is crucial for standardizing the agricultural land market order and enhancing agricultural land management efficiency. In rural China, farmer social network relationships exhibit a hierarchical structure, which manifests as differences in contract choices during land transfers. Specifically, the closer the relationship, the lower the level of caution in land transfers, and the more inclined farmers are to choose oral contracts. Conversely, the more distant the relationship, the higher the level of caution. For example, when the parties involved in a transfer have close kinship ties (e.g., relatives), the level of trust between them is higher, their caution is lower, and the role of an oral contract in safeguarding the parties’ interests is stronger, making farmers more inclined to choose oral contracts. A similar trend is observed when the parties have close geographical ties (e.g., individuals from the same village or neighboring villages).

This study addresses the issue of oral agreements dominating land transfers, aiming to promote the choice of written contracts. We explore how social relationships influence the choice of oral agreements and analyze the impact of trust and education level on the relationship between geographical ties, kinship ties, and farmer preference for oral agreements. This in-depth analysis of the influence of social relationships on the choice of land transfer contracts provides insights for policymakers and researchers, which should help them encourage farmers to choose written contracts and enhance land use efficiency.

Compared to related studies, the main innovations of this research are as follows. First, we construct an analytical framework to analyze the influence of kinship and geographical relationships on the choice of oral agreements, enriching research on land transfer contract selection. Second, based on embeddedness theory, we explore the impact of social network relationships on the choice of oral agreements, as well as the influence of social trust and education level on the relationship between social network relationships and the choice of oral agreements, expanding research on embeddedness theory and relational embeddedness in land transfer contract selection. Finally, the selected case study area is representative of the majority of the North China Plain, ensuring the scientific validity of our research.

## 2. Literature review and theoretical framework

### 2.1. The importance of formal written contracts in agricultural land transfer

Improving the land market order can not only enhance land use efficiency but also promote agricultural modernization and facilitate the implementation of rural revitalization strategies [4–6]. In China, the small-scale farming model not only makes it difficult for agriculture to achieve economies of scale, hindering the efficient utilization of agricultural production resources, but also severely restricts agricultural income growth [7]. Therefore, to promote large-scale land management, it is necessary to ensure the flexible flow of land resources, provide stable expectations for both parties in transactions, and reduce transaction uncertainty [8]. This approach can also drive agricultural income growth [9]. However, the development of non-agricultural industries and the advancement of urbanization have brought challenges to the land market. With the transfer of rural labor and the increase in the proportion of farmer non-agricultural income, Rural hollowing-out is intensifying, and the demand for agricultural land transfer is constantly increasing. Simultaneously, disputes over agricultural land transfers are gradually increasing. In the process of agricultural land transfers, due to incomplete information among farmers when reaching agreements, it is difficult for them to avoid all potential issues based on bounded rationality. For example, when either party believes that the expected cost of breaching the contract is less than the expected benefit, breaching becomes profitable, and it is inevitable that some parties will breach contracts in the pursuit of profit. Therefore, external protection mechanisms have a significant impact on the effective execution of contracts. However, the implementation of systems such as law, mortgage, trust, and arbitration relies on clear written contracts. Because oral agreements struggle to define the rights and obligations of both parties involved in a transfer, they cannot impose sufficient constraints regarding contract enforcement or adequate penalties for breaches. Therefore, although oral agreements incur lower costs compared to written contracts, they also have lower breach costs and binding force, leading to higher risks in agricultural land transfers. It is evident that encouraging farmers to choose written contracts is crucial for protecting the interests of both parties involved in a transfer and for establishing a standardized agricultural land market. However, according to existing research, in China’s land transfer market, even with government guidance, people still prefer to enter into oral agreements. Currently, more than half of agricultural land transfer contracts are oral [10].

### 2.2. Factors influencing the choice of agricultural land transfer contracts

To encourage farmers to choose written contracts when transferring land, scholars have explored various factors influencing contract selection from different perspectives.

Compared with other agricultural product markets, China’s agricultural land transfer market encompasses more social relationships such as geographical ties, kinship, and professional connections. North and Williams pointed out that transaction costs are a market failure phenomenon resulting from the interaction of human factors and transaction environment factors [11–12]. Transaction costs are the reason for changes in social institutions and can be used to explain the logic of institutional development and evolution. Therefore, in China, the choice of agricultural land transfer contracts may be influenced by social relationships. Existing research indicates that most Chinese farmers are social beings with a strong attachment to land and that rural social relationships can influence agricultural land transfers. The degree of association between transaction entities can constrain the choice of agricultural land transfer contracts. The lower the degree of association, the higher the transaction cost, and the more farmers tend to choose written contracts [13]. A strong social network helps farmers build trust, thereby reducing transaction risks and alleviating concerns regarding contract risks when both parties enter into agreements, which encourages farmers to opt for oral contracts [14]. It is evident that in agricultural land transfers, the stronger the farmer social relationships, the lower the transaction risks, and the more inclined farmers are to choose oral agreements.

### 2.3. Impact of trust on contractual choice in agricultural land transfers

Trust, as a risk-taking behavior, is strongly correlated with risk because trusting someone is equivalent to bearing the risk they introduce [15]. In agricultural land transfers, the trust between farmers and transferees, partners, and other stakeholders can strengthen farmer confidence and sense of security, reduce the risk of farmers breaching contracts, and make them more inclined to choose oral agreements [16]. A well-established land property rights system not only safeguards the order of the agricultural land market and reduces transaction risks in land transfers, encouraging farmers to choose written contracts, but also mitigates the negative impact of social relationships on farmer preferences for written contracts, thereby promoting their selection. Furthermore, the governance of agricultural land transfer contracts requires not only the practitioners of land transfers to take action but also government oversight. Supervision by village cadres can reduce the risks associated with agricultural land transfers and encourage farmers to opt for written contracts [17].

### 2.4. Impact of education level on contractual choice in agricultural land transfers

Furthermore, from the perspective of cognitive behavioral theory, farmer contract signing is influenced by their contract cognition [18]. In agricultural land transfers, the reputation mechanism achieves its effectiveness by exerting a punitive effect [19]. Reputation has a constraining and normative effect on farmers, where those with higher education levels generally possess better information literacy, have a greater understanding of laws and regulations, and exhibit stronger awareness of contract compliance. They also have enhanced capabilities in rights protection, contract fulfillment, and conflict resolution. Therefore, farmers with higher education levels typically have better reputations, and the corresponding punitive effect of reputation loss is more pronounced.

In summary, we can infer that social network relationships, trust, and education level may impact the choice of agricultural land transfer contracts. Exploring the influence of these factors enriches previous research, provides insights for researchers studying the factors affecting agricultural land transfer contract selection, and enables policymakers to formulate more effective policies when regulating the agricultural land transfer market.

### 2.5. Theoretical framework

The impact of social networks on economic behavior is one of the core issues in contemporary social sciences. In his seminal work “The Strength of Weak Ties,” Granovetter proposed that while many people search for jobs through formal channels such as job advertisements or career fairs, the reality is that most individuals secure positions through informal channels, particularly recommendations from friends and relatives [20]. Granovetter emphasized that “weak ties” in social networks often provide more extensive information resources than “strong ties,” especially when bridging different social circles, meaning such ties play a significant role in economic behavior.

However, as scholars have delved deeper into the applicability of social networks in different social contexts, particularly in relationship-oriented societies like China, it has been found that strong ties also play a crucial role in economic activities. Bian pointed out that especially in rural areas of China, kinship and geographical relationships have a decisive influence on social interactions and economic behavior [21]. Under the framework of “differential mode of association” in rural Chinese society, social behaviors are profoundly influenced by close-knit connections such as familial ties and neighborhood relationships. Kinship and geographical relationships not only constitute the core components of farmers’ social network ties but also serve as critical bonds in economic interactions.

**Fig 1:**
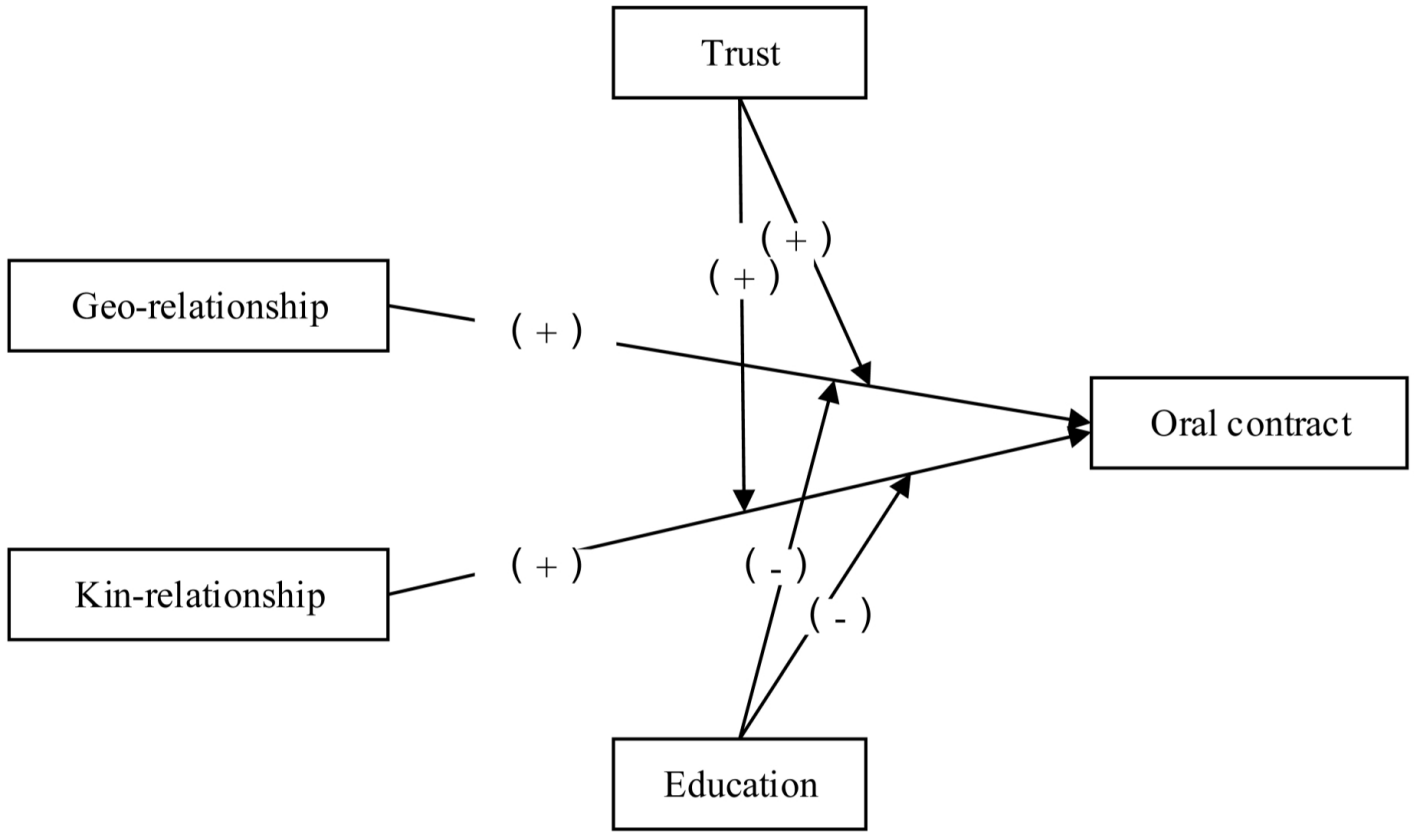
Analytical framework of the influence of geographical and kinship relationships on farmer choices of oral contracts.

Fei’s theory of “differential order” further elucidates the unique social network structure of Chinese society. Fei argues that Chinese society places particular emphasis on the social functions of kinship and geographical relationships, which is especially prominent in rural areas [22]. In rural settings, kinship relationships encompass multi-layered connections such as family members, relatives, and clans, providing farmers with strong social capital that enables mutual support in economic activities, reduces transaction costs, and enhances transaction stability. Geographical relationships based on geographical proximity and village social structures foster close interactions among farmers. This dynamic makes farmers more inclined to rely on oral agreements rather than formal written contracts in economic activities such as land transfers.Drawing on the preceding theoretical analysis, Fig 1 illustrates the analytical framework through which geographical and kinship ties influence farmers’ choices of oral contracts.

In rural China, land transfers, as a significant economic activity, are deeply influenced by social networks, particularly kinship and geographical relationships. The form of a land transfer contract is not merely a legal framework for a transaction but also a reflection of social relationships. The “emotional binding force” within kinship relationships often leads farmers to prefer relatives as trading partners during land transfers, as these relationships entail higher levels of trust and require fewer legal constraints during transactions. Oral agreements established through kinship ties typically rely on mutual understanding and interdependence to ensure transaction execution. This emotional connection reduces the supervision costs of transactions and diminishes the need to rely on formal legal frameworks.

In contrast, geographical relationships strengthen trust and cooperation among neighbors through a sense of community. In the process of land transfers, farmers often choose to transact with other farmers within the same village, as connections within the same geographical area are closer and the level of trust and interdependence is higher. Geographical relationships provide a relatively stable social safety net, particularly in the event of land transfer disputes, where traditional mediation mechanisms and interpersonal trust networks within the village can often reduce the transaction costs associated with legal procedures.

Based on the theoretical framework outlined above, we propose the following hypotheses.

H1:The closer the social network relationships, the more farmers tend to choose oral agreements for land transfers.

As a superordinate concept, social network relationships encompass the two subordinate dimensions of kinship ties (qinyuan) and geographical proximity (diyuan). To test the generalizability of Hypothesis H1, the abstract ‘social network relationships’ in H1 were operationalized into the measurable and observable dimensions of ‘kinship ties’ and ‘geographical proximity’, leading to the formulation of two sub-hypotheses: H1a (kinship ties) and H1b (geographical proximity).

H1a:The closer the kinship relationships, the more farmers tend to choose oral agreements for land transfers.

H1b:The closer the geographical relationships, the more farmers tend to choose oral agreements for land transfers.

If both H1a (kinship ties) and H1b (geographical proximity) are empirically supported (i.e., closer kinship ties and geographical proximity promote the choice of oral agreements), this would provide robust empirical support for the validity of H1, demonstrating that social networks facilitate the selection of oral agreements.

After proposing that “social networks significantly influence farmers’ contract choices (main hypothesis H1),” we further examine how this influence varies across different contexts. First, we explore the moderating role of social trust in contract selection. Social trust can be divided into two types: interpersonal trust and institutional trust. Higher interpersonal trust can reduce transaction costs and encourage farmers to choose oral agreements for land transfers. In rural China, the level of trust among farmers is relatively high, especially in kinship and neighborly relationships, which facilitates the formation of informal contracts. Institutional trust helps stabilize expectations regarding agricultural land property rights risks, enhancing farmer confidence in land transfers and influencing contract selection. Therefore, we propose the following hypothesis:

H2:The stronger the level of social trust among farmers, the greater the influence of social network relationships on contract selection.

Additionally, the education level of farmers also plays a significant moderating role in contract selection. Lin’s “social resources” hypothesis posits that resources embedded in social relationship networks are more critical than information itself [23]. For farmers, social networks are not only venues for information exchange but also provide social capital and resources that enhance their economic behavior capabilities. In the process of land transfers, farmers with higher education levels typically possess stronger legal awareness, information acquisition abilities, and decision-making skills. As a result, they are more inclined to choose written contracts, as such contracts offer stronger legal protection and reduce potential future disputes and risks. Compared with less-educated farmers, those with higher education levels are better able to understand the advantages of written contracts and do not rely entirely on the trust derived from kinship and geographical relationships. Therefore, we propose the following hypothesis.

H3:The higher the education level of farmers, the weaker the influence of social network relationships on contract selection.

## 3. Data sources, model specification, and variable selection

### 3.1. Data sources

The data used in this study came from a survey on “Agricultural Land Transfer Contract Choices” conducted by the research team in 2024 among grain-growing households in Qingyuan District, Gaobeidian City, and Dingxing County of Baoding City in Hebei Province. The research team selected Baoding City in Hebei Province for three reasons. First, Baoding City is located in the North China Plain in the hinterland of the Xiong’an New Area, connecting Beijing, Tianjin, and Shijiazhuang, and is 140 km away from Beijing. Its grain output accounts for one-fifth of that of Hebei Province, making Baoding’s food security crucial for the coordinated development of the Beijing–Tianjin–Hebei region and the Beijing–Xiong’an–Baoding area. Second, the organic matter content of arable land in Baoding City is higher than the average level in Hebei Province, with the average quality grade of arable land ranking in the upper-middle level of the Huang–Huai-Hai region. As of January 2025, Baoding City constructed 5.4 million mu of high-standard farmland, accounting for 79% of the city’s permanent basic farmland, indicating significant agricultural production potential and obvious regional advantages. Third, to protect the quality of arable land, Baoding City established 100 municipal-level arable land quality monitoring points by 2025, achieving a 100% safe utilization rate of agricultural land, reflecting the municipal government’s strong emphasis on compliant agricultural land management.

To ensure the consistency and representativeness of the questionnaire, the research team adopted random sampling and stratified sampling methods during data collection, which can be divided into two stages. In the first stage, the research team randomly selected Qingyuan District, Gaobeidian City, and Dingxing County as the survey locations. Based on the proportion of towns and villages in these three districts and counties, seven towns and streets were randomly selected in Gaobeidian City, 10 townships were randomly selected in Dingxing County, and 10 townships were randomly selected in Qingyuan District. In each town (street) of Gaobeidian City, four villages were randomly selected, while in each township of Qingyuan District and Dingxing County, two villages were randomly selected. From each village, three grain-growing households that had transferred land were randomly chosen for face-to-face questionnaire surveys, resulting in a total of 648 sample households.

The data obtained from this survey include information on household heads and their family characteristics, as well as the transfer of contracted land. A total of 648 questionnaires were completed. After excluding questionnaires with significant missing data and outliers, 636 valid questionnaires were obtained, yielding a questionnaire validity rate of 98.15%.

All data in this study underwent anonymization processing, fully complying with the provisions of Article 32(2) of the Ethical Review Measures for Life Sciences and Medical Research Involving Humans issued by The State Council of the People’s Republic of China on February 8, 2023. Therefore, this research shall be exempt from ethical review pursuant to current Chinese regulations.

### 3.2. Model specification

When transferring land, farmer economic activities are embedded in social relationships, and the strength of geographical and kinship ties between farmers and transferees can significantly influence contract choices. To analyze the impact of geographical and kinship relationship strength on contract selection, we defined contract choice as the dependent variable, and education level, general trust, specific trust, trust in village cadres, and trust in policies as moderating variables. In this study, we used IBM SPSS Statistics 27.0.1 to specify benchmark regression, moderating effect, and endogeneity test models using the logit model. For robustness checks, both logit and probit models were employed to explore the factors influencing farmer choices of agricultural land transfer contracts. The benchmark regression and moderating effect models are specified as follows.


$$\text{Contract}={\text{a}}_{\text{0}}+{\text{a}}_{\text{1}}\text{Geo}+{\text{a}}_{\text{2}}\text{Kin}+{\text{a}}_{\text{3}}\text{Control}+\epsilon_{\text{1}}$$ (1)

$$\begin{array}{cc} \text{Contract}= & \;{\text{b}}_{\text{0}}+{\text{b}}_{\text{1}}\text{Geo}*\text{Edu}+{\text{b}}_{\text{2}}\text{Kin}*\text{Edu}+{\text{b}}_{\text{3}}\text{Geo}*\text{Gen}+{\text{b}}_{\text{4}}\text{Kin}*\text{Gen}+{\text{b}}_{\text{5}}\text{Geo}*\text{Spe}+{\text{b}}_{\text{6}}\text{Kin}*\text{Spe}\\ & +{\text{b}}_{\text{7}}\text{Geo}*\text{Vil}+{\text{b}}_{\text{8}}\text{Kin}*\text{Vil}+{\text{b}}_{\text{9}}\text{Geo}*\text{Gov}+{\text{b}}_{\text{10}}\text{Kin}*\text{Gov}+{\text{b}}_{\text{11}}\text{Control}+\epsilon_{\text{2}} \end{array}$$ (2)

Here, ${\text{a}}_{\text{0}}\;$ and ${\;\text{b}}_{\text{0}}\;$ represent the intercepts, ${\text{a}}_{\text{1}}\;$ and ${\text{b}}_{\text{1}}\;$ represent the sample regression coefficients, Contract represents the type of contract chosen by the farmer, Geo represents the strength of geographical relationships, Kin represents the strength of kinship relationships, Edu  represents the education level of the household head, Gen  represents general trust, Spe  represents specific trust, Vil represents trust in village cadres, Gov represents trust in policies, Control represents control variables such as age and gender, $\epsilon_{\text{1}}\;\;\text{is}\;$ a random disturbance term, and $\epsilon_{\text{2}}$ represents unobservable factors following a standard normal distribution.

### 3.3. Variable selection

Adhering to the principles of accurately measuring variables, to ensure the comprehensibility of survey questions and feasibility of data collection, we explore factors that may influence the choice of land transfer contracts, including the personal and family characteristics of the household head and the status of contracted land transfers. By considering these variables, our research can identify multiple factors affecting the choice of agricultural land transfer contracts, providing a deeper understanding of the mechanisms through which relational embeddedness influences contract selection.

#### 3.3.1. Explained variable.

Drawing on existing research [24], the explained variable selected for this study is the “type of agricultural land transfer contract,” which is measured by the question “What type of contract did you sign for the agricultural land transfer?” If the farmer signed a written contract, it was coded as 0, whereas an oral contract was coded as 1.

#### 3.3.2. Explanatory variables.

Drawing on existing research [25], the explanatory variables selected for this study are the strength of geographical relationships and the strength of kinship relationships. These values were measured through the following two questions in the questionnaire: “What is the residential distance between you and the land transferee?” and “What is the social relationship between you and the land transferee?” Although residential distance is technically a continuous variable, daily interaction frequencies in rural acquaintance societies exhibit sharp “jumps” across the 0–1 km, 1–3 km, and 3–5 km bands rather than a smooth decline. Entering the raw continuous distance into the model risks distortion from outliers and makes it difficult to capture non-linear effects. Discretizing the variable preserves the monotonic “closer-stronger” relationship, facilitates coefficient interpretation, and enables subgroup heterogeneity tests. Therefore, geographical relationship strength is coded on a 1–5 scale as follows: 1 = distance > 10 km, 2 = 5–10 km, 3 = 3–5 km, 4 = 1–3 km, 5 = < 1 km. Similarly, kinship relationship strength is coded 1–5, where 1 = strangers or no kinship, 2 = ordinary villagers or neighbors within the same group, 3 = distant relatives or close friends, 4 = close relatives or very good friends, 5 = immediate family members.

#### 3.3.3. Moderating variables.

Based on the theoretical mechanism analysis presented earlier, the moderating variables selected for this study are education level and trust. Trust includes two aspects: interpersonal trust and institutional trust. Interpersonal trust is further divided into specific trust and general trust, while institutional trust includes trust in policies and trust in village cadres. Referring to existing research, “years of education” was used to measure education level. Specific trust was measured as “believing that relatives and friends will not breach contracts, violate rules, or engage in extensive farming practices when cultivating the land.” General trust was measured as “believing that unfamiliar people or strangers will not breach contracts, violate rules, or engage in extensive farming practices when cultivating the land.” Trust in village cadres was measured as “believing that village cadres can prevent transferees from breaching contracts, violating rules, or engaging in extensive farming practices.” Trust in policies was measured as “believing that agricultural land transfer policies can prevent transferees from breaching contracts, violating rules, or engaging in extensive farming practices.” All trust variables were measured on a 1–5 Likert scale, with the economic interpretation as follows: 1 = strongly distrust, 2 = somewhat distrust, 3 = neutral, 4 = somewhat trust, 5 = strongly trust.

#### 3.3.4. Control variables and instrumental variables.

To ensure the accuracy and reliability of our research results, we refer to previous studies and control for other factors that may influence the choice of agricultural land transfer contracts, constructing a comprehensive framework of influencing factors. The selected control variables include household head characteristics (age, gender, years of agricultural experience), family characteristics (number of family members working outside the home, health level, presence of village cadres), and contracted land characteristics (area of contracted land, status of land rights certification, land disputes, soil fertility). Furthermore, since farmers’ socioeconomic behaviors are profoundly influenced by the common characteristics of their villages, residents within the same village exhibit high degrees of spatial agglomeration and social homogeneity in terms of social networks, interaction frequency, and residential patterns. As a result, village-level averages are strongly correlated with individual values. After controlling for village fixed effects, the variation in village-level averages primarily stems from distributional differences in relationship strength among households within the village, rather than inherent village-level characteristics. Such distributional differences demonstrate negligible correlation with unobserved factors affecting individual contract choices, thereby satisfying the exogeneity condition. Based on these considerations, to address potential endogeneity issues in the model, we employ the village-level averages of kinship relationship strength and geographical relationship strength as instrumental variables. For each farmer in the sample, we exclude their own data and compute the arithmetic mean of kinship and geographical relationship strength scores of all other farmers in the village to obtain the instrumental variable value, referred to as the “village-level average kinship and geographical relationship strength.” The descriptive statistics of the sample are presented in Table 1.

**Table 1:** Descriptive statistics.

Variable Name	Variable Definitions	Average		Std.	Min	Max
Explained variable						
Kinship	Relationship between transferee and farmers (farthest = 1, nearest = 5)	4.01		1.095	1	5
Regulated variable						
Education	Years of education	9.14		2.717	5	16
General trust	Degree of trust (very skeptical = 1, trust very much = 5)	3.74		1.263	1	5
Special trust	Degree of trust (very skeptical = 1, trust very much = 5)	3.60		1.304	1	5
Trust of village cadres	Degree of trust (very skeptical = 1,trust very much = 5)	3.53		1.287	1	5
Government trust	Degree of trust (very skeptical = 1,trust very much = 5)	3.15		1.329	1	5
Control variable						
Personal characteristics of head of household						
Age	Age of head of household	46.61		9.422	24	62
Gender	Gender of head of household (woman = 0, male = 1)	0.87		0.339	0	1
Agricultural production experience	Agricultural production years	21.97		13.185	4	49
Family characteristics						
migrant	Number of migrant workers	1.81		0.789	0	4
Traffic	Have a car = 1, No car = 0	0.75		0.432	0	1
Health	If a family member has chronic disease = 1, if not = 0	0.73		0.446	0	1
Women	Number of women	1.89		0.885	0	5
Village	If there are village cadres among family members, relatives and friends = 1, if not = 0	0.31		0.463	0	1
Characteristics of contracted land						
Contracted	Mu of contracted land	4.25		1.236	3	8
Disputes	Occurrence of land disputes (happened = 0, never happened = 1)	0.92		0.487	0	1
Certificate	If the certificate of title is perfect = 1, if not = 0	0.82		0.381	0	1
Fertility	Fertility degree (very barren = 1, very fertile = 5)	4.27		0.562	1	5

## 4. Results

### 4.1. Baseline model

The baseline regression results are presented in Table 2. Without considering regional differences, the estimation results based on the logit model lead to the following conclusions.

**Table 2:** Benchmark regression results.

Variable	Ⅰ	Ⅱ	Ⅲ	Ⅳ	Ⅴ
Intensity of geopolitical relations		0.234*** (0.1105)		0.537*** (0.161)	0.376*** (0.241)
Kinship strength			0.398*** (0.075)	0.299*** (0.146)	0.428*** (0.209)
Education	−0.125 (0.042)				−0.259 (0.076)
General trust	0.562*** (0.083)				0.398*** (0.114)
Special trust	0.315*** (0.044)				0.424*** (0.095)
Trust of village cadres	−0.415*** (0.063)				−0.366*** (0.096)
Government trust	−0.108*** (0.049)				−0.243*** (0.088)
Control variable	control	control	control	control	control
Constant	−0.240*** (0.340)	−0.196*** (0.415)	−0.176*** (0.347)	−0.159*** (0.496)	−0.146 (0.385)
N	636	636	636	636	636
LR chi^2^ (2)	134.85	116.56	83.74	79.51	250.82
Prob > chi^2^	0.000	0.000	0.000	0.000	0.000
Pseudo R2	0.342	0.183	0.151	0.242	0.192

Note: ***, **, and * indicate significance at the 1%, 5%, and 10% levels, respectively. Robust standard errors are in parentheses.

For Model I, a baseline regression was conducted on the control variables, revealing that education level is insignificant, whereas the remaining control variables are significant at the 1% level.

For Models II to IV, as the strengths of geographical relationships and kinship relationships are sequentially added as independent variables, one can see that the strengths of geographical relationships and kinship relationships are positively correlated with farmers’ choice of oral agreements, and both are significant at the 1% level.

In all directions, after sequentially introducing the strength of geographical relationships, kinship relationships, and control variables in the baseline regression, we find that the closer the geographical and kinship relationships, the more farmers tend to choose oral agreements. The direction and significance of the impact of geographical and kinship relationship strength on farmers’ choice of oral agreements exhibit no significant changes. The influence of geographical and kinship relationships on farmers’ choice of oral agreements aligns with our hypotheses, and the robustness of the model estimation results is strong, validating hypotheses H1, H1a, and H1b. Additionally, in Model V, the odds ratios for the strength of geographical relationships and kinship relationships are 1.075 and 1.249, respectively. Therefore, for every unit increase in the strength of geographical and kinship relationships, the odds of farmers choosing oral agreements increase by 7.5% and 24.9%, respectively. These results not only emphasize the influence of social network relationships on farmers’ choice of oral agreements but also highlight the role of strengthening community consciousness and emotional bonds in promoting farmers’ preference for oral agreements.

### 4.2. Endogeneity test

Because directly using the logit model for analysis may lead to errors in regression results and potential endogeneity issues, we construct an instrumental variable model to address these possible endogeneity problems. A valid instrumental variable must satisfy two conditions: it must be highly correlated with the endogenous explanatory variable and weakly correlated with the disturbance term. In this study, the strength of geographical relationships and kinship relationships, as well as the choice of agricultural land transfer contracts, may be simultaneously influenced by unobserved omitted variables. Additionally, potential measurement errors could also lead to estimation biases. Therefore, to address potential endogeneity issues in the model, we construct two instrumental variables: the average strength of geographical relationships within the same village and the average strength of kinship relationships within the same village, followed by a two-stage regression test.

In the regression results presented in Table 3, the coefficients for the average strength of geographical relationships within the same village and the average strength of kinship relationships within the same village are 0.853 and 0.614, respectively, both of which are significant at the 1% level. The F-test values are much larger than the critical values, indicating that the selected instrumental variables are strongly correlated with the explanatory variables, eliminating weak instrument issues and confirming the validity of the instrumental variables. The coefficients for the strength of geographical relationships and kinship relationships are 0.276 and 0.391, respectively, both of which are significant at the 1% level, revealing a positive correlation with farmers’ choice of oral agreements. Therefore, after considering endogeneity, the influence of geographical and kinship relationship strength on the choice of agricultural land transfer contracts aligns with the baseline regression results. This instrumental variable test not only verifies the reliability of the baseline regression conclusions but also supports the main research findings.

**Table 3:** Endogeneity test.

Variable	Intensity of geo-relations	Oral contract	Kin-strength	Oral contract
Average intensity of geographical relations in the same village	0.853*** (19.56)			
Intensity of geo-relations		0.276*** (4.49)		
Mean value of kinship intensity in the same village			0.614*** (18.67)	
Kin-strength				0.391*** (6.72)
Control variables	Control	Control	Control	Control
Cragg–Donald Wald F	72.873***	68.514***	136.375***	105.189***
Adj R2	0.074	0.119	0.352	0.191
Constant	0.273*** (0.064)	0.679** (0.104)	0.254*** (0.098)	0.481** (0.127)
N	636	636	636	636

Note: ***, **, and * indicate significance at the 1%, 5%, and 10% levels, respectively. Robust standard

errors are in parentheses.

### 4.3. Moderating effect analysis

To explore the influence of education level and social trust on the relationship between social network relationships and choice of agricultural land transfer contracts, we introduce interaction terms for education level and social trust with geographical and kinship relationships into the model sequentially. The results in Table 4 reveal the impact of education level and trust on the relationship between geographical and kinship relationships, and farmer choice of oral agreements. The findings are summarized below.

**Table 4:** Moderating effect analysis results.

Variable	Ⅰ	Ⅱ	Ⅲ
Intensity of geopolitical relations × Education	−0.310*** (0.0735)		−0.151*** (0.0604)
Kinship strength × Education	−0.250 (0.020)		−0.294 (0.035)
Intensity of geopolitical relations × General trust		0.213** (0.037)	0.158** (0.089)
Kinship strength × General trust		0.175* (0.063)	0.230* (0.071)
Intensity of geopolitical relations × Special trust		0.104* (0.105)	0.136* (0.091)
Kinship strength × Special trust		0.243*** (0.082)	0.281*** (0.063)
Intensity of geopolitical relations × Government trust		−0.125*** (0.038)	−0.176*** (0.051)
Kinship strength × Government trust		−0.103*** (0.061)	−0.120*** (0.073)
Intensity of geopolitical relations × Trust of village cadres		−0.213*** (0.081)	−0.308*** (0.070)
Kinship strength × Trust of village cadres		−0.257*** (0.078)	−0.212*** (0.094)
Control variable	Control	Control	Control
Constant	−0.138** (0.329)	−0.084 (0.216)	−0.144 (0.164)
N	636	636	636
LR chi2 (2)	294.36	328.15	371.62
Prob > chi2	0.000	0.000	0.000
Pseudo R2	0.217	0.197	0.261

Note: ***, **, and * indicate significance at the 1%, 5%, and 10% levels, respectively. Robust standard errors are in parentheses.

To explore the influence of education level and social trust on the relationship between social network relationships and the choice of agricultural land transfer contracts, we introduce interaction terms for education level and social trust with geographical and kinship relationships into the model sequentially. The results in Table 4 reveal the impact of education level and trust on the relationship between geographical and kinship relationships and farmers’ choice of oral agreements. The findings are summarized below.

In the results of Model I, education level is negatively correlated with the relationship between geographical relationships and farmers’ choice of oral agreements, and this correlation is significant at the 1% level.

In the results of Model II, general trust is positively correlated with the relationship between the strength of geographical relationships and farmers’ choice of oral agreements, significant at the 5% level. General trust is also positively correlated with the relationship between the strength of kinship relationships and farmers’ choice of oral agreements, significant at the 10% level. Specific trust is positively correlated with the relationship between the strength of geographical relationships and farmers’ choice of oral agreements, significant at the 10% level, and is also positively correlated with the relationship between the strength of kinship relationships and farmers’ choice of oral agreements, significant at the 1% level. Policy trust is negatively correlated with the relationship between the strength of geographical relationships and farmers’ choice of oral agreements, significant at the 10% level, and is also negatively correlated with the relationship between the strength of kinship relationships and farmers’ choice of oral agreements, significant at the 1% level. Trust in village cadres is negatively correlated with the relationship between the strength of geographical relationships and farmers’ choice of oral agreements, significant at the 10% level, and is also negatively correlated with the relationship between the strength of kinship relationships and farmers’ choice of oral agreements, significant at the 1% level. Therefore, the higher the general trust and specific trust, the stronger the influence of geographical and kinship relationships on farmers’ choice of oral agreements. Conversely, the higher the policy trust and trust in village cadres, the weaker the influence of geographical and kinship relationships on farmers’ choice of oral agreements.

In summary, the direction and significance of the impact of education level and trust on the relationship between social network relationships and farmers’ choice of oral agreements remain consistent, indicating strong robustness in the model estimation results. The influence of education level on the relationship between social network relationships and farmers’ choice of oral agreements aligns with hypothesis H3.

However, the impact of social trust on the relationship between social network relationships and farmers’ choice of oral agreements exhibits heterogeneity. Higher interpersonal trust strengthens the relationship between social network relationships and farmers’ choice of oral agreements, whereas higher institutional trust weakens this relationship. This heterogeneity may arise for two reasons. First, trust in interpersonal interactions can enhance the strength of social relationships and reinforce the trust derived from social networks. Therefore, strengthening interpersonal trust amplifies the relationship between social networks and farmers’ choice of oral agreements. Second, the credibility of policies and the leadership of village cadres help reduce farmers’ reliance on relatives and friends. Institutional trust can weaken the trust derived from social networks, meaning that strengthening institutional trust diminishes the relationship between social networks and farmers’ choice of oral agreements. These findings not only demonstrate the influence of education level and trust on the relationship between social network relationships and farmers’ choice of oral agreements but also highlight the complexity of factors affecting agricultural land transfer contract selection.

### 4.4. Robustness tests

To verify the robustness of the baseline regression results, we conducted two sets of tests: alternative variable tests and subsample analysis. In the first set of tests, we replaced the original variables “strength of kinship ties” and “strength of geographical ties” with “social expenditure” and “distance between the farmer’s residence and the transferee’s residence,” respectively. Here, social expenditure is defined as the total cash or in-kind expenses incurred by a household over the past year to maintain interactions with relatives (including both consanguineal and affinal kin), covering gifts for weddings, funerals, and festivals, ceremonial offerings, banquet hospitality, and shared ritual expenses. The survey item was phrased as: “In the past 12 months, how much did your household spend in total on gifts and hospitality for relatives?” In the context of rural China, such expenditures represent one of the most quantifiable and normatively regulated forms of kinship interaction. Higher values indicate more frequent kinship exchanges and denser kinship networks. In the subsample analysis, we excluded extreme or atypical observations by partitioning the sample into three groups: low kinship strength (scores 1–2), medium kinship strength (score 3), and high kinship strength (scores 4–5).

Table 5 reports the robustness results. In the alternative-measure test, both social expenditure and residential distance are positively and significantly associated with the choice of oral contracts at the 1% level, consistent with the baseline expectations. In the subsample analysis, the positive and significant effect of kinship strength on the likelihood of choosing an oral contract is replicated across low-, medium-, and high-strength groups, again at the 1% level. These findings corroborate the credibility of the baseline estimates, reinforce the impact of the key explanatory variables, and further demonstrate the reliability of our conclusions.

**Table 5:** Robustness test results.

Variable	Replace Variable	Low genetic strength	Moderate genetic strength	Higher genetic strength
Amount of human expenditure	0.3143*** (0.3182)			
Distance between farmer and transferee permanent residences	0.1658*** (0.0649)			
Intensity of geopolitical relations		0.1761*** (0.0426)	0.1549*** (0.0917)	0.1810*** (0.0628)
Kinship strength		0.2901*** (0.0586)	0.2805*** (0.0193)	0.1632*** (0.0274)
Control variables	Control	Control	Control	Control
Constant	−0.3569*** (0.3249)	−0.3962*** (0.5530)	−0.4038*** (0.4016)	−0.3527*** (0.5871)
N	636	249	102	285
LR chi2 (2)	164.35	210.87	286.49	195.26
Prob > chi2	0.0000	0.0000	0.0000	0.0000
Pseudo R2	0.2612	0.1704	0.1918	0.1573

Note: *** and ** indicate significance at the 1% and 5% levels, respectively. Robust standard errors are in parentheses. The control variables are the same as those in Table 2.

## 5. Discussion

### 5.1. Main findings

In rural China, agricultural land is a personalized asset for farmers. Land transfers among farmers are mostly based on “relational logic” and “survival logic,” which are often influenced by traditional beliefs and emotional factors [26]. Many farmers have a strong attachment to their land, and even if they are willing to transfer it, they prefer to do so within kinship and geographical networks [27]. For example, studies have shown that 88% of land transfers occur within the same village, indicating that geographical and kinship relationships play a significant role in land transfers [28,29]. During the land transfer process, geographical and kinship relationships help establish trust among farmers, forming an informal governance mechanism that reduces transaction risks and distrust of strangers. As a result, farmers are more inclined to transfer land to relatives, neighbors, and other acquaintances within the same village. Because the pre-, during-, and post-contract transaction costs are lower when dealing with relatives and neighbors, and the contract signing process is simpler and more flexible, land transfers based on geographical and kinship relationships often rely on oral agreements. Relevant studies have also pointed out that geographical and kinship relationships promote farmers’ choice of oral agreements.

This study focuses on the impact of social network relationships on the choice of agricultural land transfer contracts. Our findings are summarized as follows: (1) Geographical and kinship relationships significantly promote farmers’ preference for oral agreements, highlighting the limiting role of these relationships in standardizing the agricultural land transfer market. (2) Education level weakens the influence of social networks on farmers’ choice of oral agreements, meaning that more educated farmers are less likely to rely on informal agreements. (3) Interpersonal trust strengthens the relationship between social networks and oral agreement preference, whereas institutional trust weakens this effect, as farmers with higher institutional trust are more inclined to adopt written contracts.

Regarding the influence of education level on the relationship between social networks and farmers’ choice of oral agreements, there may be two reasons for this influence. First, written contracts have higher legal validity, better protecting the rights and interests of both parties and reducing transaction risks. Farmers with higher education levels are more aware of the protective role of written contracts and are therefore more inclined to choose them. Second, although oral agreements lack legal protection, are prone to disputes, and are less stable, they have lower transaction costs. Farmers with lower education levels find oral agreements easier to understand and more convenient to establish, making them more likely to choose oral agreements. Regarding the influence of interpersonal trust on the relationship between social networks and farmers’ choice of oral agreements, there may be two factors at play. First, specific trust strengthens the role of personal relationships, and this trust mechanism can effectively reduce transaction costs. Second, general trust reduces farmers’ wariness toward most people during land transfers, thereby promoting their choice of oral agreements. Regarding the influence of institutional trust on the relationship between social networks and farmers’ choice of oral agreements, there may be two reasons. First, trust in policies makes farmers more confident in the legal protections provided by written contracts and the effectiveness of policy implementation. Second, trust in village cadres makes farmers believe that the government can effectively regulate the land transfer market, safeguard their rights, and prevent disputes, thereby encouraging them to choose written contracts.

### 5.2. Limitations and future prospects

Although this study analyzed the impact of geographical relationships, kinship relationships, education level, and trust on the choice of agricultural land transfer contracts, there are still some limitations. First, factors beyond geographical and kinship relationships, such as cognitive, institutional, and structural embeddedness, also influence the choice of agricultural land transfer contracts. This study only considered the impact of geographical and kinship relationships, leaving insufficient exploration of other embeddedness factors. To address this limitation, future research could incorporate other embeddedness factors into the analytical framework for agricultural land transfer contract selection, examining their influence to gain a deeper understanding of how social embeddedness affects contract choices. Second, over time, changes in rural economic development levels, population structures, and social relationships will also alter the influence of relational embeddedness on agricultural land transfer contract choices. The data collected in this study were obtained over a short period and do not reflect the impact of relational embeddedness at different time points. To address this limitation, future research could adopt multi-time-point analyses to compare effects at different times, providing a more comprehensive analysis of how relational embeddedness influences agricultural land transfer contract choices.

By analyzing the influence of social network relationships on the choice of agricultural land transfer contracts, we revealed pathways that can be considered to promote farmer selection of written contracts. First, this study highlights the role of social network relationships in encouraging farmers to choose oral agreements. To promote the selection of written contracts, it is necessary to strengthen the construction of laws and regulations, encourage diversified farming practices, and facilitate the transfer of rural labor. These measures can enhance the institutional safeguards for written contracts, reduce farmer dependence on land, and weaken the promoting effect of geographical and kinship relationships on farmer preference for oral agreements. Second, this study underscores the role of education level in promoting farmer choice of written contracts. Therefore, it is essential to increase investment in rural education, enrich farmer knowledge structures, and establish an information service network system for agricultural markets. These steps can improve farmers’ ability to access information and strengthen their understanding of written contracts, thereby enhancing the role of education in promoting their selection. Finally, this study highlights the role of interpersonal trust in promoting farmer choice of oral agreements and institutional trust in promoting their choice of written contracts. To encourage the selection of written contracts, it is necessary to enhance farmers’ awareness of standardized operations and strengthen economic rationality, thereby weakening the promoting effect of interpersonal trust on oral agreements. Additionally, clarifying the management authority of village cadres and improving land dispute mediation mechanisms can strengthen the role of institutional trust in promoting farmer choice of written contracts. Furthermore, this study not only explains the role of social network relationships in promoting farmer choice of oral agreements but also demonstrates the impact of social network relationships on economic behavior, emphasizing the social functions of geographical and kinship relationships. This study also sheds light on the influence of social trust and education level on farmer choice of oral agreements, enriching the understanding of the reasons behind farmer preference for such agreements.

By analyzing the influence of social network relationships on the choice of agricultural land transfer contracts, this study highlights pathways to promoting farmer selection of written contracts, providing valuable references for policymakers. These insights can help formulate targeted governance strategies for agricultural land transfer contracts, ensuring an efficient, fair, and stable land transfer market, improving land use efficiency, reducing land disputes, and maintaining rural order. Additionally, this study enriches the research paradigm on agricultural land transfer contract selection, offering valuable insights for future research.
